# Characterization of Bacterial Communities in Breeding Waters of *Anopheles darlingi* in Manaus in the Amazon Basin Malaria-Endemic Area

**DOI:** 10.1007/s00248-019-01369-9

**Published:** 2019-04-15

**Authors:** Louise K. J. Nilsson, Marta Rodrigues de Oliveira, Osvaldo Marinotti, Elerson Matos Rocha, Sebastian Håkansson, Wanderli P. Tadei, Antonia Queiroz Lima de Souza, Olle Terenius

**Affiliations:** 1 Department of Cell and Molecular Biology, Microbiology, Uppsala University, P.O. Box 596, 751 24 Uppsala, Sweden; 2 Department of Ecology, Swedish University of Agricultural Sciences (SLU), P.O. Box 7044, 750 07 Uppsala, Sweden; 3 Programa de Pós-graduação em Biotecnologia e Recursos Naturais da Amazônia, Universidade do Estado do Amazonas, Manaus, AM 69065-001 Brazil; 4 Department of Molecular Biology and Biochemistry, University of California, 3205 Mc-Gaugh Hall, Irvine, CA 92697 USA; 5 Programa de Pós-graduação em Biotecnologia, Universidade Federal do Amazonas, Manaus, AM Brazil; 6 Laboratório de Malária e Dengue, Instituto Nacional de Pesquisas da Amazonia, Manaus, AM 69011-970 Brazil; 7 Uppsala BioCenter. Department of Molecular Sciences, Swedish University of Agricultural Sciences (SLU), P.O. Box 7025, 750 07 Uppsala, Sweden; 8 Faculdade de Ciências Agrárias, Universidade Federal do Amazonas (FCA/UFAM), Manaus, AM 69080-900 Brazil

**Keywords:** Microbiota, Amazonas, Malaria, *Anopheles darlingi*, Mosquito, 16S rRNA

## Abstract

The microbiota in mosquito breeding waters can affect ovipositing mosquitoes, have effects on larval development, and can modify adult mosquito-gut bacterial composition. This, in turn, can affect transmission of human pathogens such as malaria parasites. Here, we explore the microbiota of four breeding sites for *Anopheles darlingi*, the most important malaria vector in Latin America. The sites are located in Manaus in the Amazon basin in Brazil, an area of active malaria transmission. Using 16S rRNA gene sequencing by MiSeq, we found that all sites were dominated by Proteobacteria and Firmicutes and that 94% of the total number of reads belonged to 36 operational taxonomic units (OTUs) identified in all sites. Of these, the most common OTUs belonged to *Escherichia*/*Shigella*, *Staphylococcus*, and *Pseudomonas*. Of the remaining 6% of the reads, the OTUs found to differentiate between the four sites belonged to the orders Burkholderiales, Actinomycetales, and Clostridiales. We conclude that *An*. *darlingi* can develop in breeding waters with different surface-water bacteria, but that the common microbiota found in all breeding sites might indicate or contribute to a suitable habitat for this important malaria vector.

**Electronic supplementary material:**

The online version of this article (10.1007/s00248-019-01369-9) contains supplementary material, which is available to authorized users.

## Introduction

The Amazon tropical rainforest contains ~ 25% of the world’s terrestrial biodiversity [1]. Along with the many species of plants, insects, mammals, and birds, microorganisms abound and their diversity is still unknown. In recent years in many parts of the world, extensive research has been performed on the diversity and function of environmental microbial communities and the microbiota associated with humans, animals, and plants [2–6]. It is now known that these microorganisms contribute to nutrient cycling, food webs, detoxification, and the wellbeing of plants, animals, and ecosystems [7]. One example where the microbiota plays an important role is in the development of mosquito larvae because mosquito larvae feed on microorganisms and in particular on bacteria in their breeding water [8, 9]. Several studies have even shown that bacteria are indispensable for mosquito development. For example, as early as 1935, Rozeboom [10] showed that *Aedes aegypti* could not develop in bacteria-filtered water. Touré et al. [11] observed large larval mortality in water treated with antibiotics and Coon et al. [12] showed that axenic larvae failed to develop past first instar. Furthermore, bacteria have been shown to both attract [13, 14] and repel [15] gravid mosquitoes to potential breeding sites, suggesting they direct ovipositing females. Besides affecting oviposition and development of larvae, the bacteria in the breeding water might also impact the microbiota of the adult mosquitoes, as adult mosquitoes have been shown to contain gut bacteria found in their breeding water [12, 16, 17]. Moreover, the gut bacteria in adult mosquitoes have been correlated to pathogen survival in the mosquitoes. For example, a *Chromobacterium* sp. (*Csp_P*) has been shown to increase resistance to infection of both Dengue virus in *Ae. aegypti* and the malaria-causing agent *Plasmodium falciparum* in *Anopheles gambiae* [18], and an *Enterobacter sp*. (*Esp_Z*) has been shown to increase resistance to *P. falciparum* in *An. gambiae* [19]. Genetic modification of mosquito gut bacteria has also been suggested as a tool to prevent malaria transmission by producing anti-parasitic molecules in the mosquito gut [20].

The mosquito *An. darlingi* is the principal malaria vector in the Amazon basin [21], responsible for 875,000 cases of malaria per year on the American continent [22]. While research has been conducted on the microbiota of breeding waters of malaria vectors from the Old World, in rice paddies, semi-natural and natural habitats in Kenya [23–25], breeding sites in Iran [26], natural habitats in Cameroon [17], and domestic water-storage containers in India [27], very little is known about the microorganisms associated with the breeding waters of malaria vectors on the South American continent. As a matter of fact, the number of publications concerning bacteria associated with *An. darlingi* is very limited. Thus far, one study by Rejmankova et al. [28] investigated the number of bacteria classified as cocci or rods associated with larval habitats of four species of *Anopheles* in Belize. They showed that overall cocci were most common and there was some difference in number of cocci and rods between some of the habitats with different *Anopheles* species. However, the bacteria were not classified further. In a pilot study on *An. darlingi* adults, Terenius et al. [29] obtained 56 16S rRNA gene sequences from six host-seeking mosquitoes. All of these belonged to Gammaproteobacteria, where sequences closely related to *Enterobacter*, *Pantoea*, *Aeromonas*, and *Pseudomonas* were most abundant. In a recent study of the feces of *An*. *darlingi*, culture-based methods identified five genera of bacteria belonging to the classes Gammaproteobacteria and Bacilli [30]. A recent study on gut bacteria in two Colombian malaria vectors, *An. darlingi* and *An. nuneztovari*, showed that the most important determinants of gut bacteria composition was developmental stage followed by geographical location [31]. These two determinants were more important than mosquito species or adult feeding status.

*Anopheles* larvae are filter feeders that use their head brushes to feed on particles found in the surface microlayer (SML) [32]. The SML has chemical and biological properties that differ greatly from the water a few centimeters below surface. The *Anopheles* larvae are not selective feeders, but typically, the size of the particles ingested is less than 50 μm [32], which means that bacteria belong to the food commonly ingested. In this study, our objective was to characterize the bacterial community composition in the SML of *An. darlingi* breeding waters. To do this, we used MiSeq sequencing of 16S rRNA gene amplicons from four *An. darlingi* breeding sites in Manaus, Brazil. While different habitats may result in location-driven variability in bacterial composition, it has also been shown that in the same type of habitats, the presence of certain groups of bacteria is correlated with the presence of mosquito larvae (see, e.g., [27]). We therefore hypothesized that either certain species or larger taxonomic groups of bacteria (up to community level) and the conditions they indicate would be similar in all sites as the sites are comparable in size and characterized by large abundances of *An. darlingi* larvae.

## Methods

### Water Sampling

Surface water was collected from four water bodies in the Manaus municipality (Table 1, Online Resource Fig. A.1a), an area of active malaria transmission. The four collection sites located in Manaus are permanent *Anopheles* breeding sites as determined by the Vigilância Epidemiológica da Secretaria Municipal de Saúde de Manaus [Epidemiological Surveillance of the Municipal Health Secretariat in Manaus]. At each of the four collection sites, samples were obtained from four equidistant sub-sites, approximately 5 m from each other, on the lake/dam/fish tank perimeter (Online Resource Fig. A.1b.). Surface water samples (900 mL) were collected in the morning at 8:30 AM using a hand-dipper and stored on ice in sterile flasks for transportation. In the laboratory, each water sample was filtered through three overlaid filters (filter paper Whatman grade 4, and millipore membranes of 0.45 μm and 0.22 μm; Online Resource Fig. A.1b). The retained material was eluted from each filter in 2 mL of distilled and autoclaved water and centrifuged for 12 min at 10,000*g*. The supernatant was discarded and the pellet DNA extracted. Twelve samples were DNA extracted per site, originating from three filters from each of the four sub-sites per collection site (Online Resource Fig. A.1b). Thus, in total, 48 samples were DNA extracted.

**Table 1:** Geographic location and characteristics of the *Anopheles darlingi* breeding sites where water was collected. Maps and additional information about the sites are found in Supplementary Fig. A.1 and Supplementary information A.1

Site	Location	Characteristics	GPS coordinates		Collection date (Month/year)
			Latitude (S)	Longitude (W)	
1	Puraquequara–Portela	Lake with small fish, shaded edges	03° 03. 081′	059° 53. 594′	01/2013
2	Puraquequara–Estrada do Brasileirinho	Dam, no fish, vegetation in margins	03° 01. 190′	059° 54. 700′	03/2013
3	Puraquequara–Sítio do Carlão	Fish tank, no vegetation	03° 02. 770′	059° 52. 874′	04/2013
4	AM 010–Extension of Sítio Canarinho	Lake with fish, vegetation in margins	02° 53. 730′	059° 54. 969′	04/2013

### DNA Extraction of Water Samples and 16S rRNA Gene Amplification by PCR

To obtain the total DNA from the water samples, DNA extractions were performed by first lysing the cells by heat shock of the pellets from the filtrates. Then, the innuPREP Plant DNA kit (Analytik Jena) was used following the manufacturer’s protocol. The handling of biological material from the Amazon region is strictly regulated. To avoid bringing any material (including genomic DNA) from Manaus to our laboratory facilities in Sweden, we performed a first-step PCR in Manaus. In order to not affect the downstream processes, we started with primers outside the target region V3-V4. Recovered DNA was thus dissolved in 20 μL nuclease-free water (Invitrogen) and the bacterial 16S rRNA genes were amplified by PCR using illustra PuReTaq Ready-To-Go PCR Beads (GE Healthcare) and the primers 27F (5′-AGAGTTTGATCMTGGCTCAG-3′) [33] and 1100R (5′-AGGGTTGCGCTCGTT-3′) modified from reference [34]. The PCR program had an initial denaturation at 95 °C for 5 min, followed by 30 cycles of 94 °C for 1 min, 56 °C for 1 min, 72 °C for 2 min, followed by a final extension at 72 °C for 10 min. Amplicon production and size were verified by electrophoresis in a 1% agarose gel. In our experience, lack of PCR amplification products results in no sequences. Therefore, negative controls obtained from DNA extraction and/or PCR amplification were not further processed and were not sequenced. The amplicons obtained were sent to Uppsala, Sweden, and used for the library preparation.

### Library Preparation and MiSeq Sequencing

From the 16S rRNA gene PCR products obtained from the water samples, the V3-V4 region (*Escherichia coli* position 341–805) was amplified by a two-step PCR method. In the first step, the general bacterial primers 341F (5′-CCTACGGGNGGCWGCAG-3) and 805R (5′-GACTACHVGGGTATCTAATCC-3) [35] were used, which match approximately 90% of all bacterial sequences and cover all phyla in the Ribosomal Database Project release 10.25. Each DNA sample was individually PCR-amplified with illustra PuReTaq Ready-To-Go PCR Beads (GE Healthcare) by initial denaturation at 95 °C for 5 min, followed by 20 cycles of 95 °C for 40 s, 53 °C for 40 s, 72 °C for 1 min, followed by a final extension at 72 °C for 7 min. The PCR products were analyzed by microchip electrophoresis using the MCE-202 MultiNA (Shimadzu) and diluted in nuclease-free water (Invitrogen) to a concentration of 0.1–1 ng/μL. In the second step, 1 out of 50 flanking barcode sequence pairs was added to each sample (to be able to run samples in parallel) [36] using the same conditions as above, but only for 10 cycles of iteration. The PCR products were analyzed by microchip electrophoresis as before and pooled together, 50 differently barcoded samples per pool and 60 ng per sample. In total, 6 pools of 50 samples each were created. Of the 300 samples, 48 samples belonged to this project and were randomly distributed over the 6 pools to avoid pool- sequencing bias. Each pool was purified using illlustra GFX PCR DNA and Gel Band Purification Kit (GE Healthcare) and eluted in 50 μL nuclease-free water (Invitrogen). The pools were sent to the SNP&SEQ Technology Platform in Uppsala, Sweden (www.sequencing.se) for further processing and sequencing. Sequencing libraries were prepared from ~ 10 ng of DNA using the ThruPLEX-FD Prep Kit (R40048-08, Rubicon Genomics) according to the manufacturer’s instructions. The libraries were purified using AMPure XP beads and the quality evaluated using the 2200 TapeStation system (Agilent Technologies) and the D1000 Analysis ScreenTape assay. The adapter-ligated fragments were quantified by qPCR using the Library quantification kit for Illumina (KAPA Biosystems) on a StepOnePlus instrument (Applied Biosystems/Life technologies) prior to cluster generation and sequencing. The pooled DNA samples were paired-end sequenced with 300 bp read length on the MiSeq system (Illumina) using the v3 chemistry according to the manufacturer’s protocols. The raw MiSeq reads are available in the ENA database hosted by EBI under the accession number PRJEB25809.

### Bioinformatics Analysis

The paired-end reads were assembled and demultiplexed using Mothur (version 1.36.1) [37] resulting in 741,779 reads keeping sequences with fewer than two base differences between the primer portion of the read and the primer. Further analyses were performed by USEARCH (version 8.1.1861) [38]. Reads were filtered to remove low-quality reads using a maximum expected error threshold of one. The remaining sequences were de-replicated using full-length matching. Clustering of operational taxonomic units (OTUs) was performed using UPARSE [39] with a minimum identity of 97% and discarding singletons and chimeras. To make the OTU table, the reads before quality filtering and removal of singletons were mapped to the OTUs using a minimum identity of 0.97 to the representative sequence. Reads that were classified as chloroplasts were removed from the dataset manually in Microsoft Excel, leaving 416,420 reads in 154 OTUs for analysis. Reads from the three different water filters from the same site and sub-site were added together and treated as one sample in downstream analyses, yielding 16 samples (four sub-site samples for each of the four breeding sites). The taxonomic annotation of the OTUs was performed using the UTAX RDP train set 15 and a pre-trained taxonomy confidence file for the sequence length 500. Taxonomical annotation was performed with a confidence threshold of 0.9.

### Data Analysis

All data analysis in R was performed using the R-software (version 3.3.3) [40] in R-studio (versions 1.1.383 and 1.1.456) [41]. To visualize the distribution of the reads among the samples, a bar chart was created in the R-package “ggplot2” [42] (Online Resource Fig. A.2). To normalize the reads between samples, the reads per OTU in the OTU table were converted into percentage of reads in each sample that belonged to each OTU. From rarefaction curves, it was seen that many samples reached a plateau suggesting that adequate sequencing depth was obtained for these samples. However, some samples did not level off suggesting insufficient sequencing depth, which can lead to an underestimation of bacterial diversity in these samples, though this was mainly seen for site 1, which despite this had the highest observed alpha diversity (Online Resource Fig. A.3). Rarefaction curves were created in the R-package “vegan” [43]. To summarize and compare the bacterial community composition in the different *An. darlingi* breeding sites, bar charts showing the distribution of bacterial phyla, classes, and families were created. The distribution of bacteria at the different taxonomic levels was visualized in bar charts produced in the R-package “ggplot2” [42]. An in-house Python script was used to extract the families present in the samples. The most common OTUs in all of the breeding sites were then identified and visualized by a bar chart produced by the same method as above. To look for differences in alpha diversity in the breeding sites, the OTU table was first rarefied to 10,073 reads per sample (based on the sample with the fewest reads) using the R-package “vegan” [43]. Observed and estimated (Chao1) species richness was calculated using the R-package “phyloseq” [44]. To illustrate the alpha diversity, box plots were created using the function “plot” in R. To visualize the similarities in bacterial community composition between the sites and the sub-sites (beta-diversity), non-metric multidimensional scaling (NMDS) plots were created. These were made using the R-package “vegan” [43] on the Bray-Curtis dissimilarity index based on OTU abundance in the samples. To explore which OTUs differed between the bacterial communities in the breeding sites (beta-diversity), an indicator species analysis was performed using the R-package “labdsv” [45].

### Statistics

For comparison of alpha diversity between the sites, analysis of variance (ANOVA) was first performed in R. This was based on normal distribution and homogeneity of variance of the samples, identified in R by Shapiro-Wilk’s test and Bartlett’s test, respectively. Following a statistically significant ANOVA result, pairwise comparisons using two-tailed *t* tests with pooled standard deviation and *p* value adjustment method Holm were performed in R. To compare the differences between the bacterial community compositions in the breeding sites, permutation-based analysis of variance (PERMANOVA) was performed with 10^6^ permutations and the beta-diversity measuring method Bray-Curtis in the R-package “vegan” [43]. Following a statistically significant PERMANOVA result, pairwise PERMANOVA was performed using the R-package “RVAideMemoire” [46] based on the Bray-Curtis dissimilarity matrix with 10^6^ permutations and *p* value adjustment method Benjamini and Hochberg.

## Results

### Bacterial Community Composition

**Fig. 1:**
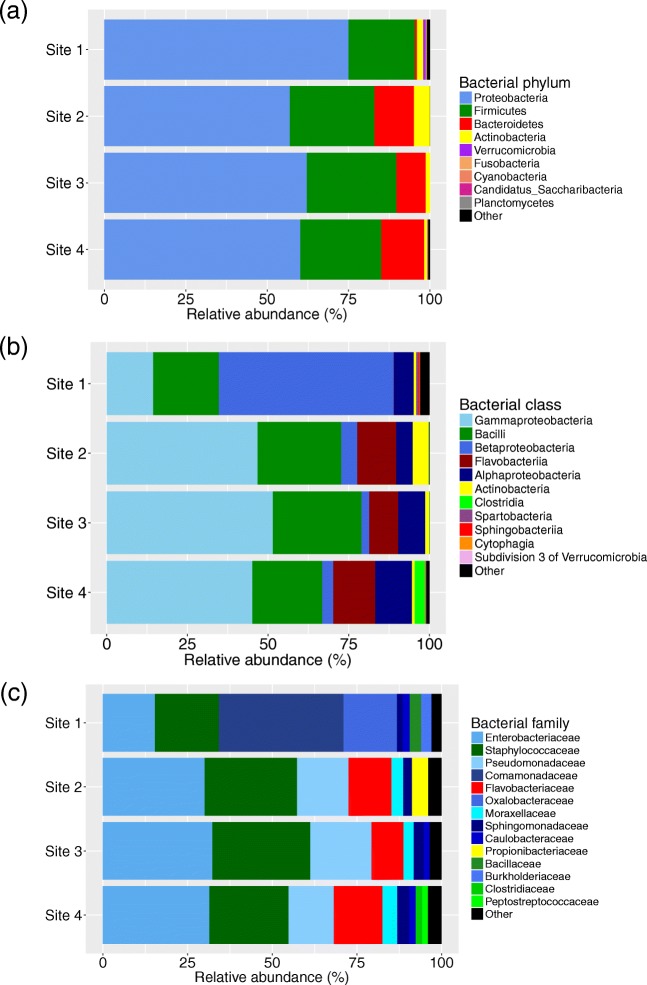
Bacterial community composition in different *Anopheles darlingi* breeding sites in Manaus. **a** Bacterial community composition at phylum level, “Other” = unknown phylum. **b** Bacterial community composition at class level. Only classes making up > 0.1% in any sample are named, other classes present are clustered as “Other” together with unknown classes. **c** Bacterial community composition at family level. Only families making up > 1% in any sample are named, other families present are clustered as “Other” together with unknown families

After sequencing the hypervariable V3-V4 region of the bacterial 16S rRNA gene and bioinformatics processing of the water samples from four *An. darlingi* breeding sites in Manaus, a total of 416,420 reads were left in 154 OTUs (the number of reads per sample was between 10,073 and 64,585, with an average of 26,026 reads per sample). The OTUs were classified into nine phyla with the top three phyla making up 98% of all the reads, Fig. 1a. Taken together, the data show that on average, 63% of all the reads belonged to Proteobacteria making it the most common phylum. The second and third most common phyla were Firmicutes and Bacteroidetes, making up 25% and 9%, respectively. The nine phyla were divided into 14 classes with the top three classes making up 79% of the reads (Fig. 1b). The top three classes were Gammaproteobacteria, Bacilli, and Betaproteobacteria, which made up 42%, 24%, and 12%, respectively. In total, 38 families were identified. The bacterial families Enterobacteriaceae, Staphylococcaceae, and Pseudomonadaceae were most common, making up 27%, 24%, and 12% of the reads, respectively (Fig. 1c). The separation of sequences for each sub-site is shown in Online Resource Fig. A.4.

**Fig. 2:**
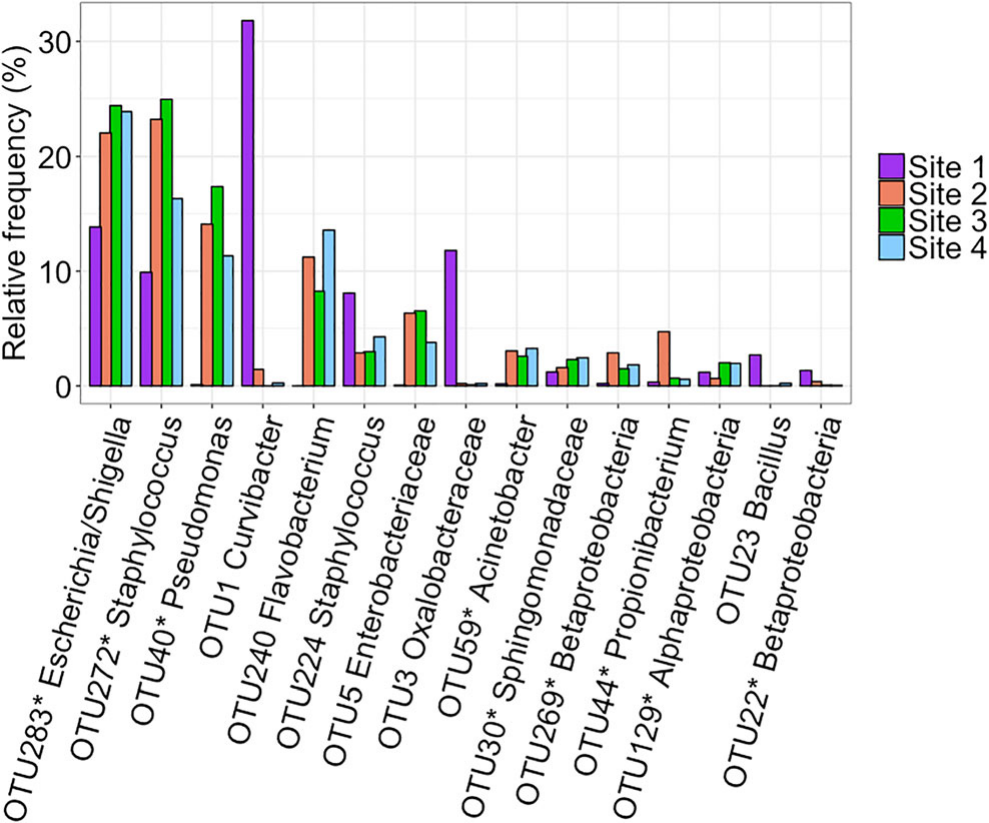
Common operational taxonomic units (OTUs) found in all *Anopheles darlingi* breeding sites in Manaus. The top 15 most common OTUs are shown together with the identified taxonomy at the lowest available level. *OTUs that were found in all sub-sites

Only 36 out of all the identified 154 OTUs were common in all breeding sites (Online Resource Fig. A.5), and even fewer were identified in all sub-sites in all breeding sites (10 out of 154 OTUs). However, 94% of all the reads belonged to one of those 36 common OTUs. The relative frequencies of the most common OTUs found in all breeding sites show that the top three OTUs are *Escherichia/Shigella* (OTU283), *Staphylococcus* (OTU272), and *Pseudomonas* (OTU40, Fig. 2).

### Alpha Diversity

**Fig. 3:**
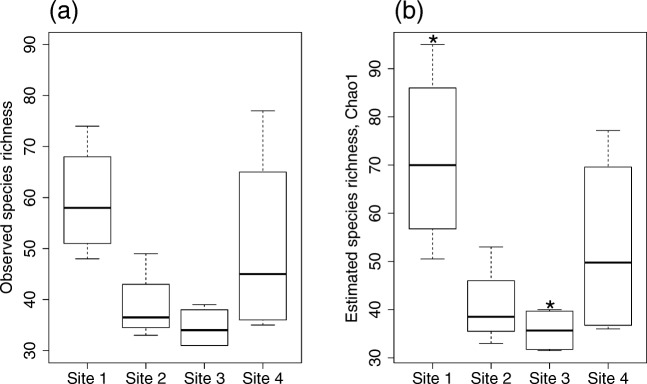
Alpha diversity in *Anopheles darlingi* breeding sites in Manaus. **a** Observed species richness and **b** estimated species richness, Chao1. *Difference in alpha diversity based on pairwise comparisons between breeding sites (*p* value < 0.05). The samples were rarefied to 10,073 reads per sample before analysis

Species richness, observed and estimated (Chao1), was compared between the four breeding sites (Fig. 3). Both indices were included to investigate both the actual number of OTUs observed and the number of OTUs estimated to exist from the abundance data by Chao1. Chao1 was included to account for OTUs that could be present in the breeding sites but not observed due to under sampling and too shallow sequencing depth. Observed species richness tended to differ between sites 1 and 3, but the difference was not statistically significant (Fig. 3a). However, when the species richness was estimated by Chao1, a statistically significant difference in alpha diversity appeared between breeding sites 1 and 3 (*p* = 0.029; Fig. 3b). On average, 46 OTUs were observed per site and 52 OTUs were estimated per site by the Chao1 method.

### Bacterial Community Composition in Sub-sites

**Fig. 4:**
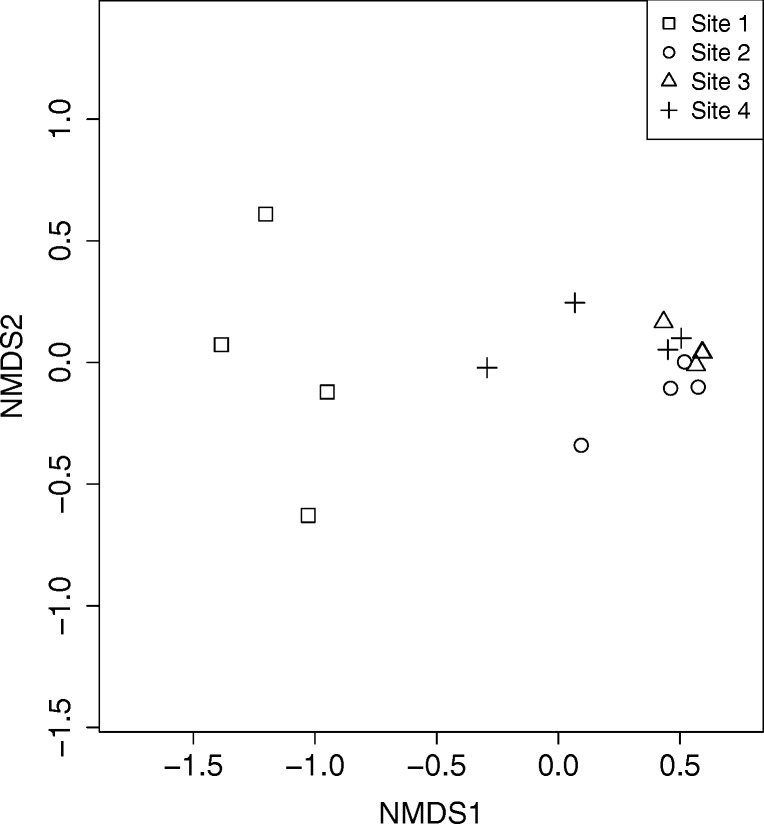
Non-metric multidimensional scaling (NMDS) plots of water samples. The plots are based on all bacterial operational taxonomic units (OTUs) in the samples and separated by *Anopheles darlingi* breeding site. In each breeding site, four sub-sites were sampled. The points for two sub-sites from site 3 are overlaid. Stress value: 0.074

To investigate the similarity of the bacterial community composition in the sub-sites from the four breeding sites, the OTUs from the sub-sites were compared, Fig. 4. When classifying the sub-sites according to which main breeding site they belonged to, it was found that the bacterial communities vary significantly between sites based on PERMANOVA (*p* = 5 × 10^−6^). Water samples that were taken from different sub-sites within the same main breeding site seemed to contain similar bacteria. When the sub-sites were compared by pairwise PERMANOVA based on the main breeding site they belonged to, it was found that all four sites were significantly different in bacterial community composition from each other except for sites 3 and 4 that were not significantly different, Table 2.

**Table 2:** Pairwise PERMANOVA comparing the beta diversity in *Anopheles darlingi* breeding sites in Manaus. Based on Bray-Curtis dissimilarity matrix with 10^6^, i.e. 1 million permutations and *p* value adjustment method Benjamini and Hochberg. *P* values for each comparison are shown

	Manaus 1	Manaus 2	Manaus 3
Manaus 2	0.034	–	–
Manaus 3	0.034	0.034	–
Manaus 4	0.034	0.034	0.057

### Discriminatory OTUs

To investigate which bacterial OTUs were driving the differences between the breeding sites, an indicator value analysis selecting discriminatory OTUs was performed according to Dufrene and Legendre [47] (Table 3). The OTUs with a significant indicator value for a specific breeding site are here referred to as “discriminatory OTUs”. Indicator values range from 0 to 1 where the indicator value 1 means that an OTU is found in all samples in a defined group and that the specific OTU only is found in the defined group. Thirteen OTUs with high indicator values (> 0.7) were identified as discriminatory OTUs for breeding site 1, many of which were identified as species within the order Burkholderiales. Sites 2–4 had only two or three identified discriminatory OTUs each. For sites 2 and 3, three of the five OTUs were associated with the order Actinomycetales. For site 4, the OTUs were associated with the order Clostridiales (Table 3).

**Table 3:** Discriminatory OTUs identified by indicator species analysis for water from different *Anopheles darlingi* breeding sites in Manaus

Site	OTU	Indval	*p* value	Freq.	Taxonomy^a^	Top hit^b^
1	OTU142	1.00	0.006	4	p:Proteobacteria	Uncultured *Leptothrix*-99%
	OTU16	0.998	0.005	5	p:Verrucomicrobia, c:Spartobacteria	Uncultured bacterium FukuN106–98%
	OTU38	0.966	0.009	6	p:Actinobacteria	Uncultured Micrococcineae −97%
	OTU3	0.958	0.007	12	p:Proteobacteria, c:Betaproteobacteria, o:Burkholderiales, f:Oxalobacteraceae	
	OTU1	0.949	0.003	8	p:Proteobacteria, c:Betaproteobacteria, o:Burkholderiales, f:Comamonadaceae, g:*Curvibacter*	
	OTU17	0.921	0.005	6	p:Proteobacteria, c:Betaproteobacteria, o:Burkholderiales, f:Burkholderiaceae, g:*Polynucleobacter*	
	OTU9	0.864	0.004	11	p:Proteobacteria, c:Betaproteobacteria, o:Burkholderiales, f:Oxalobacteraceae	
	OTU52	0.750	0.032	3	p:Proteobacteria	–
	OTU91	0.750	0.018	3	p:Proteobacteria, c:Betaproteobacteria	Uncultured *Limnohabitans*-99%
	OTU24	0.750	0.032	3	p:Bacteroidetes, c:Cytophagia, o:Cytophagales, f:Cytophagaceae, g:*Flectobacillus*	
	OTU94	0.750	0.027	3	p:Proteobacteria, c:Betaproteobacteria	*Deefgea*-100%
	OTU22	0.733	0.022	16	p:Proteobacteria, c:Betaproteobacteria	Uncultured Comamonadaceae-97%
	OTU160	0.702	0.029	7	p:Proteobacteria, c:Betaproteobacteria, o:Burkholderiales	*Curvibacter*-99%
2	OTU44	0.748	0.007	16	p:Actinobacteria, c:Actinobacteria, o:Actinomycetales, f:Propionibacteriaceae, g:*Propionibacterium*	
	OTU273	0.642	0.023	7	p:Actinobacteria, c:Actinobacteria, o:Actinomycetales, f:Microbacteriaceae, g:*Leifsonia*	
	OTU269	0.448	0.002	16	p:Proteobacteria, c:Betaproteobacteria	*Curvibacter delicatus* strain N30–99%
3	OTU83	0.565	0.028	12	p:Actinobacteria, c:Actinobacteria, o:Actinomycetales, f:Nocardiaceae, g:*Rhodococcus*	
	OTU40	0.405	0.018	16	p:Proteobacteria, c:Gammaproteobacteria, o:Pseudomonadales, f:Pseudomonadaceae, g:*Pseudomonas*	
4	OTU315	0.744	0.039	5	p:Firmicutes, c:Clostridia, o:Clostridiales, f:Clostridiaceae_1, g:*Clostridium_sensu_stricto*	
	OTU87	0.715	0.033	6	p:Firmicutes, c:Clostridia, o:Clostridiales, f:Peptostreptococcaceae, g:*Clostridium_XI*	

*OTU* operational taxonomic unit, *Indval* indicator value, *Freq.* frequency, number of samples the OTU occurred in

^a^p, c, o, f, and g refer to the taxonomic levels phylum, class, order, family, and genus, respectively

^b^OTUs not identified to family level were compared to sequences in the database on the NCBI website using nucleotide BLAST with the representative sequence per OTU. The named bacterium (if any) with identity of 97%–100% is given

## Discussion

In this study, we explored the bacterial community composition of *An. darlingi* breeding waters by characterizing and comparing the communities of four breeding sites in Manaus, Brazil, using 16S rRNA gene amplicon sequencing. We hypothesized that bacteria and the conditions they indicate would be similar in all sites because they all are characterized by large abundance of *An. darlingi* larvae. Although we found that 94% of the total number of reads belonged to 36 OTUs identified in all sites and that Proteobacteria and Firmicutes dominated in all sites, at lower taxonomic levels the bacterial composition diverged between sites. Three sites were similar in their bacterial composition, while one site differed significantly.

Previous studies on the microbiota of *Anopheles* breeding waters have mainly investigated those of Old World malaria vectors [17, 23–27]. Comparing our results from breeding waters of a New World malaria vector with those studies show that overall the same bacterial composition at phylum level was found. The most common phyla in this study were Proteobacteria, Firmicutes, Bacteroidetes, and Actinobacteria. Proteobacteria were present in all of the above-mentioned studies. In Iran, Firmicutes was also identified in breeding sites for *An. stephensi* and *An. maculipennis* by 16S rRNA gene sequencing of isolated bacteria [26]. Three studies that use 454-pyrosequencing of 16S rRNA genes, which is a method more comparable to this study, obtained similar results. The four phyla that we identified as the most common in our breeding sites were also the most common in the surface layer of *An. coluzzii* and *An. gambiae* breeding waters in Cameroon [17]. These four phyla were also among the most common in *Anopheles* breeding waters on three Kenyan islands in Lake Victoria [25]. However, in their study Actinobacteria formed a smaller part of the total composition than in our study. Similarly, Wang et al. [24] identified the four phyla to be common in the surface layer of semi-natural breeding sites in Kenya. However, they found Cyanobacteria as the second most common phylum. In the study of domestic water-storage containers in India by Nilsson et al. [27], the same sequencing method (MiSeq) was used and the four most common phyla were the same as identified here. At class level, the bacterial composition was also similar with Gammaproteobacteria and Bacilli being among the most common in this study and also in the five studies of *Anopheles* breeding waters in the Old World. However, in the semi-natural breeding sites in Kenya, Alphaproteobacteria and Cyanobacteria were most abundant [24]. In the domestic water-storage containers in India, Alphaproteobacteria and Betaproteobacteria were also abundant [27], and on the Kenyan islands, Betaproteobacteria was most abundant [25]. This abundance of Betaproteobacteria was similar to our breeding site 1 that contained more Betaproteobacteria than Gammaproteobacteria (Fig. 1b). In a recent study by Bascuñán et al. [31] on New World *Anopheles* mosquitoes (*An. nuneztovari* and *An. darlingi*), the proportion of Gammaproteobacteria as similar in the breeding waters as compared to our data (~ 40%). However, this proportion increased during development likely reflecting the selection induced by the alkaline environment in the larval gut. At family level, the bacteria identified by Dinparast Djadid et al. [26] were Pseudomonadaceae, Moraxellaceae, Aeromonadaceae, Enterobacteriaceae, and Bacillaceae; of these all but Aeromonadaceae were identified in this study. The most common families identified by Wang et al. [24], Methylocystaceae, Cyanobacteria/FamilyII, and Acetobacteraceae, were different from the ones we identified. Taken together, the fact that by in large the same kind of bacteria are found in the breeding sites of different species of malaria mosquitoes from both the Old World and the New World suggests that, although separated by continents and several million years of evolution, the *Anopheles* mosquitoes prefer to breed in waters with similar characteristics.

Of the bacteria common to all sites in this study (36 OTUs), several of the most abundant (Fig. 2) are connected to human habitats such as *Escherichia/Shigella*, *Staphylococcus*, and *Pseudomonas* indicating strong human influence on the microbiota. This is expected, as the sites are located near human habitations. Nevertheless, these bacteria have also been shown to appear frequently in other studies on mosquitoes and their breeding waters. For example, Dada et al. [48] reported from Thailand and Laos results where high abundance of *E. coli* was strongly correlated to the presence of *Ae. aegypti* mosquitoes. However, in their follow-up study, the correlation between abundance of *E. coli* and mosquitoes was not as strong [49]. For *Pseudomonas*, Chavshin et al. [50] showed that in Iranian *An*. *culicifacies*, these were the most common bacteria and that identical *Pseudomonas* 16S rRNA gene sequences were found in samples from locations far apart indicating a strong association with *An. culicifacies*. Although not yet convincingly shown, one could expect the bacteria in the surface microlayer to elicit odors that attract female mosquitoes. *An. darlingi* is considered a highly anthropophilic species breeding in close proximity to human settlements. In this regard, we speculate that the presence of the bacteria strongly connected to humans (*Escherichia/Shigella*, *Staphylococcus*, and *Pseudomonas*) could be used by the female mosquitoes as olfactory cues of where to oviposit.

Breeding site 1 (Portela) stands out as being different from the other sites at all taxonomic levels (Fig. 1), and also has the highest alpha diversity of the four sites with a significantly higher estimated richness than site 3 (Fig. 3). This higher alpha diversity might partly explain why many more discriminatory OTUs were identified for site 1 than for the other sites (Table 3). The difference of site 1 is also seen in the NMDS plot (Fig. 4) where all sub-sites from site 1 separate from the rest. At phylum level, Bacteroidetes occurs in very small numbers in site 1, which consistently make up the third largest group in the other three sites. At a lower taxonomic level, it is seen that this group mainly is made up of Flavobacteriaceae and is also reflected in Fig. 2 where *Flavobacterium* is the fifth most common bacterium overall. One factor contributing to this difference in bacterial composition in breeding site 1 could be the collection date as this site was sampled earlier in the year than sites 2–4 (seasonal variations in bacterial composition in lakes have been observed [51]). However, the climate in Manaus from January to May 2013 showed little variability in terms of temperature and rainfall.

Several different species belonging to the order Burkholderiales were identified as descriptors of site 1. One discriminatory OTU not identified as Burkholderiales was identified as a species of *Flectobacillus* (Table 3). *Flectobacillus* (*roseus*) was recently found to be a fish pathogen [52], and as site 1 receives a stream with fish, this might explain the presence of *Flectobacillus* here. Otherwise, site 1 stands out as containing several bacterial genera previously isolated from and associated with fresh or brackish water sources (*Flectobacillus*, *Curvibacter*, and *Polynucleobacter*) [53–55]. This finding could indicate that the water at site 1 was less affected by human settlements. One of the discriminatory species for site 2, Propionibacterium, could indicate human or animal presence as it is commonly described as a commensal part of the skin microbiota in both humans and animals [56].

*An. darlingi* was abundant in the breeding sites investigated here. Although the breeding sites had different levels of vegetation and the amount of fish varied from no fish at all to the sites being fish tanks, *An. darlingi* larvae thrived in all of them. Previously, several studies have investigated environmental characteristics associated with *An. darlingi* larvae and found that they prefer large, deep, and clear water bodies, such as lakes, swamps, and rivers [21]. Also, fishponds have been shown to function as important breeding sites for *An. darlingi* [57, 58] with four times more larvae than natural water bodies [57]. Even though some environmental characteristics have been associated with *An. darlingi* larvae and *An. darlingi* was shown to be the least tolerant of three *Anopheles* species to habitat types other than their own typical habitat [59], they exist in many habitat types [60]. *An. darlingi* density has also been found to be higher in larval habitats closer to human habitations [61] and to be good at adapting to environments modified by human development [21]. This suggests that *An*. *darlingi* is opportunistic, and though it might prefer some types of habitats, it can breed in water with different types of bacteria as shown in this study.

## Conclusions

Most cases of malaria on the American continent occur in the Amazon region where *An. darlingi* is the most important vector [62]. We hypothesized that bacteria and the conditions they indicate would be similar in all locations as they all are characterized by large abundance of *An. darlingi* larvae. However, based on our findings of diverging bacterial communities, we draw the conclusion that *An*. *darlingi* can develop in breeding waters with different surface-water bacteria although the common microbiota found in all breeding sites might contribute to the suitable habitat. Bacteria in mosquito breeding-waters have been shown to affect oviposition by adult mosquitoes and development of larvae as well as being taken up by mosquitoes and forming part of their gut microbiota [8–17]. Therefore, this new information on bacteria in *An. darlingi* breeding waters could be useful in vector- and malaria-control strategies based on natural mosquito- and parasite-inhibitory effects of the bacteria, or by genetic modification of the bacteria to prevent transmission of malaria parasites.

## Electronic supplementary material

ESM 1 (DOCX 2015 kb)
