# Supplementary material for: Characterization of Bacterial Communities in Breeding Waters of Anopheles darlingi in Manaus in the Amazon Basin Malaria-Endemic Area
Source: Microb Ecol. 2019 Apr 15;78(4):781–91. doi: 10.1007/s00248-019-01369-9 (PMC6842340; doi:10.1007/s00248-019-01369-9)
Supplement: Supplementary file 1 — (DOCX 2015 kb) [file 248_2019_1369_MOESM1_ESM.docx]

**Supplementary material**

Characterization of bacterial communities in different breeding waters of *Anopheles darlingi* from the Amazon basin malaria-endemic area.

Louise K. J. Nilsson*, Marta Rodrigues de Oliveira, Osvaldo Marinotti, Elerson Matos Rocha, Sebastian Håkansson, Wanderli P. Tadei, Antonia Queiroz Lima de Souza and Olle Terenius.

*Corresponding author: [olle.terenius@icm.uu.se](mailto:olle.terenius@icm.uu.se). Department of Cell and Molecular Biology, Microbiology, Uppsala University, Box 596, SE-751 24, Uppsala, Sweden.

**SUPPLEMENTARY INFORMATION**

Supplementary information A.1. *Anopheles darlingi* breeding sites where water was collected.

**Site 1 Puraquequara - Portela**

Private property located in a forested area occupied by people living from agriculture. It is part of the metropolitan region, in the eastern part of Manaus called Puraquequara. It is an area altered by human occupation. At first there was a stream (igarapé) in the place, then little by little it was silted and took shape of a lake, where its edges became shaded, ideal for development of mosquito larvae. The National Institute of Amazonian Research (INPA) has monitored this breeding site for over a decade and collects weekly larvae and adults of *Anopheles darlingi*, the most abundant mosquito species in the area. According to INPA surveillance data, there are *Anopheles* mosquitoes throughout the year with a higher peak in the second semester (July-December). The lake receives a stream (igarapé) containing small fish. Some fish remain for long periods in the lake while others follow the stream (igarapé) to other lakes.

**Site 2 Puraquequara - Estrada do Brasileirinho**

Medium-sized dam fed by a source of water, located in an area surrounded by forest with human habitations. According to the constant monitoring by INPA, there is a great density and diversity of anophelines present together with marginal vegetation and macrophytes. At the site there is no fish breeding, however, nearby, there are some large lakes used for fish farming.

**Site 3 Puraquequara - Sítio do Carlão**

Active fish tank located near the forest with human habitations. Due to daily human activity, farming, the site does not present vegetation in the margins and has moderate ciliary forest that contributes to the shading where the *Anopheles* larvae can be found. It is characterized as a semipermanent fish nursery with influence of another larger tank that is supplied by a source of water. Larvae can be found year-round, however with a lower density compared to other breeding grounds.

**Site 4 AM 010 - Extension of Sítio Canarinho**

Natural lake located far from the urban area of Manaus. For several years, the locals have used it as a fish tank for domestic purposes. The water that feeds this breeding site comes from a lake that has its own source of water. The environmental structure is partially shaded with a forest around and vegetation in the margins, where *Anopheles* larvae develop. According to INPA, larvae can be found year-round.

**SUPPLEMENTARY FIGURES**

(a)

(b)

Fig. A.1. Collection-site information. (a) Map showing the locations of the *Anopheles* *darlingi* breeding sites 1-4 in Manaus, Brazil, where water was collected. Map created from Google maps. (b) Schematic diagram showing the sampling setup for generation of samples to extract DNA from.

Fig. A.2. Distribution of reads per sub-site after processing of raw MiSeq reads.

Fig. A.3. Rarefaction curves for each sub-site. Colored according to *Anopheles darlingi* breeding site. Step size for sample sizes = 100.

(a)

(b)

Fig. A.4. Bacterial community composition in four different *Anopheles darlingi* breeding sites in Manaus. Each site has four sub-sites 5m from each other. (a) Bacterial community composition at phylum level, “Other”=unknown phylum. (b) Bacterial community composition at family level. Only families making up >1% in any sample are named, other families present are clustered as “Other” together with unknown families.


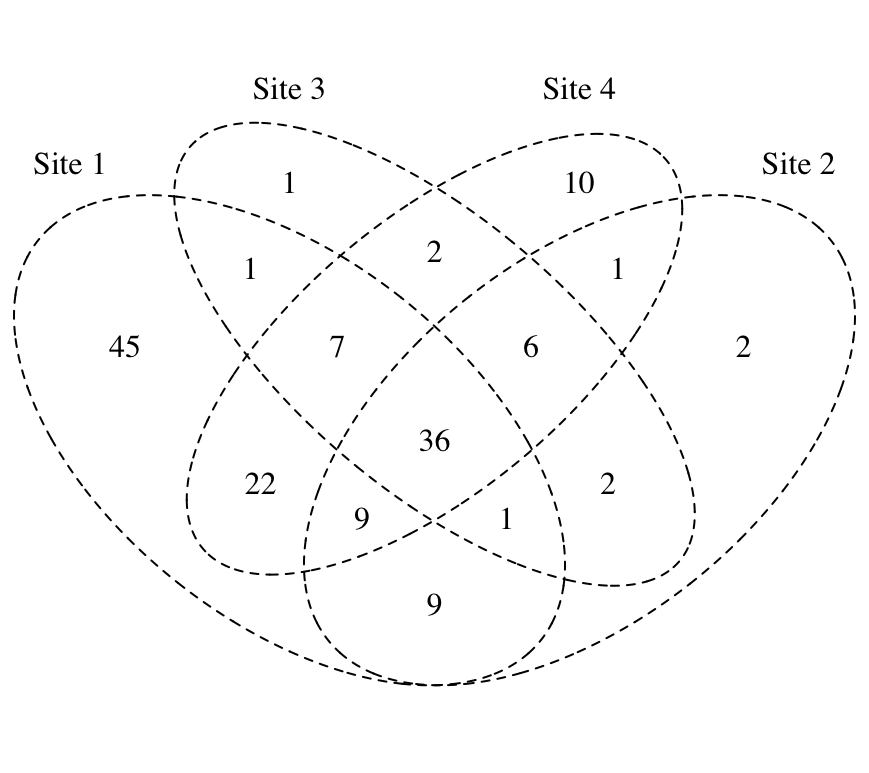


Fig. A.5. Venn diagram of the distribution of all OTUs identified. Each number corresponds to the number of OTUs found in each site or sites.
